# Lactate induced up‐regulation of KLHDC8A (Kelch domain‐containing 8A) contributes to the proliferation, migration and apoptosis of human glioma cells

**DOI:** 10.1111/jcmm.15780

**Published:** 2020-08-26

**Authors:** Xiaolong Zhu, Tianbing Chen, Hui Yang, Kun Lv

**Affiliations:** ^1^ Key Laboratory of Non‐coding RNA Transformation Research of Anhui Higher Education Institutes (Wannan Medical College) Wuhu China; ^2^ Non‐coding RNA Research Center of Wannan Medical College Wuhu China; ^3^ Central Laboratory The first affiliated hospital of Wannan Medical College Wuhu China

**Keywords:** apoptosis, glioma, KLHDC8A, lactate, proliferation

## Abstract

Glioma is a common type of malignant brain tumour with high mortality and relapse rate. However, the molecular mechanisms of glioma development have not been clarified. Differentially expressed genes in normal brain tissues and glioma tissues, low‐grade and high‐grade gliomas were screened out with GEO database analysis. We found that KLHDC8A (Kelch domain‐containing 8A) expression level was significantly increased in high‐grade glioma tissues and that high KLHDC8A expression was closely related with poor prognosis. Function assays indicated that KLHDC8A knockdown inhibited proliferation, migration and invasion, blocked the cell cycle and promoted apoptosis in glioma cells. Mechanistically, KLHDC8A regulated various functions in glioma by directly mediating Bcl2, BAX, p21, CDK2, MMP2 transcription and ERK and P38 MAPK activation. KLHDC8A overexpression enhances glioma tumorgenesis such as cell proliferation, migration and invasion. The ERK and P38 MAPK which activated by KLHDC8A overexpression could be reversed by U0126 and SB203580, respectively. Meanwhile, stimulation of lactate which produced by glycolysis is responsible for induction of KLHDC8A expression. Collectively, we demonstrated that KLHDC8A plays an important role in tumorgenesis of glioma, suggesting that it is a promising prognostic marker and a potential therapy target for the treatment of glioma.

## 1 INTRODUCTION

Glioma is the most common tumour with the highest mortality rate in the central nervous system.^1^ Glioma can be classified into three grades (II, III and IV) according to the 2016 World Health Organization (WHO) classification.^2^ Among the three grades, grade II were subdivided as low‐grade gliomas and grade III and IV were subdivided as high‐grade gliomas. The 2015 annual report of the National Central Cancer Registry of China (NCCR) shows that the morbidity and mortality of glioma patients are increasing year by year.^3^ Glioma possesses many characteristics, including highly invasive, rich blood vessels and high recurrence rate.^4^ The traditional multi‐modal therapy with surgery, radiotherapy and chemotherapy cannot effectively improve the prognosis of glioma patients. The average survival time of glioma patients is <1 year, and the number of people with 5‐year survival time is less than 5%.^5^ Therefore, clarifying the tumorigenesis of glioma is a prerequisite for seeking effective treatment measures.

KLHDC8A encodes a kelch domain‐containing protein which is up‐regulated in cancer. Kelch domain‐containing proteins belong to the Kelch superfamily which includes 63 protein‐coding genes and 3 non‐coding genes. The Kelch superfamily of proteins can be subdivided into KLHL, KBTBD and KLHDC subfamilies by the presence of a Kelch‐repeat domain.^6^ Kelch superfamilies have roles in cell migration, cell morphology, protein degradation, gene expression, cytoskeletal arrangement and extracellular communication/interaction.^7^, ^8^ Kelch family members are involved in a number of disease such as neuromuscular diseases, tumour, pseudohypoaldosteronism, autosomal dominant retinitis pigmentosa.^9^

KLHDC8A is a member of the KLHDC subfamily that consists of either Kelch domain repeats alone or with other domains such as transmembrane, Glycine rich, or Lish and CTLH domains. KLHDC subfamily proteins are reported to play role in the development of several tumours. KLHDC4 promotes NPC (Nasopharyngeal Carcinoma) oncogenesis by suppressing cellular apoptosis and may serve as a prognosis biomarker and a potential therapeutic target for NPC.^10^ Reduced expression of KLHDC8B leads to an increase in binucleated cells, thus recapitulating a hallmark of classical Hodgkin lymphoma.^11^ Previous research has shown that KLHDC8A is highly expressed in human gliomas which ΔEGFR was silenced. Knockdown of KLHDC8A let to decreased tumorigenicity in ΔEGFR‐independent ‘escaper’ tumours.^12^ However, the function and molecular mechanism of KLHDC8A in the development of gliomas are still not clear.

In this study, we detected the KLHDC8A expression in glioma tissues and analysed its relevance with the overall survival of glioma patients. We also investigated the contribution of KLHDC8A to the function of glioma cells and the underlying mechanism. Our findings provide the role of KLHDC8A in the pathogenesis of glioma and KLHDC8A may be a potential diagnostic and prognostic marker.

## 2 MATERIALS AND METHODS

### 2.1 Patients and tissue samples

Archival human glioma tissue samples and non‐glioma patient samples were obtained from Department of Neurosurgery, the first affiliated Hospital of Wannan Medical College. Samples were quickly removed at surgery and immediately divided into two parts; one part was fixed in 4% paraformaldehyde for 24 hours, paraffin embedded and used for histopathological diagnosis, and the remaining part was snap frozen in −80°C until used for RNA isolation. The use of these archival tissues in this study was approved by the Ethics Committee of the first affiliated Hospital, Wannan Medical College. All methods used in this study were performed in accordance with the relevant guidelines and regulations. Meanwhile, the informed consent was obtained from all participants and/or their legal guardians.

### 2.2 Microarray analysis

The mRNA expression array data were acquired from Gene Expression Omnibus (GEO) database (accession number is GSE4290 and GSE2223) that had been published previously.^13^, ^14^ The GSE4290 data set includes gene expression profiles from 23 normal brain tissues and 157 glioma samples (26 astrocytomas, 50 oligodendrogliomas and 81 glioblastomas). The GSE2223 data set includes gene expression profiles from 50 glial brain tumours (31 glioblastomas, 14 oligodendrogliomas and 5 astrocytomas) and 4 normal brains. The differentially expressed mRNAs between normal with tumour samples, grade II with grades III and IV were performed by GEO2R. Log_2_ (FC)>2 or >1.5 and *P*‐value < 0.05 were set as the cut‐off criteria, respectively.

### 2.3 Gene expression profiling and survival analysis

We used GEPIA2 (Gene Expression Profiling Interactive Analysis, http://gepia.cancer‐pku.cn/index.html) to analyse the gene expression data and overall survival which base on TCGA (The Cancer Genome Atlas) and GTEx (Genotype‐Tissue Expression) projects.^15^

### 2.4 Cell lines and culture

The human glioma cell lines (U87MG, U251) were used in this study. These cell lines were purchased from American Type Culture Collection (Manassas, VA). Cells were cultured in Dulbecco's Modified Eagle Medium (DMEM) (Hyclone, GE Healthcare Life Sciences) which supplemented with 10% (v/v) foetal bovine serum (FBS) (Gibco, Life Technologies) at 37°C in an atmosphere of 5% CO_2_.

### 2.5 RNA extraction and real‐time qPCR analysis

Total RNA was extracted from the brain tissues and glioma cells by using TRIzol reagent (Ambion, life technologies) as previously described.^16^ Then, the RNA was used to synthesize first strand cDNA with the RevertAid First Strand cDNA Synthesis Kit (Thermo Scientific). RT‐qPCR was conducted using QuantiNova™ SYBR^®^ Green PCR Kit (Qiagen) protocol with Bio‐Rad CXF96 PCR system (Bio‐Rad). Each sample was performed in triplicate and the expression of the target gene was normalized to GAPDH which served as internal control. The relative gene expression was calculated with the comparative cycle threshold (2^−ΔΔCt^) method. Primer sequences for qPCR: KLHDC8A Forward, TGTGACCCTGGACAACCACT; KLHDC8A Reverse, GTCGAACACGTCCATCGTCC; LDHA Forward, CAG CCCGATTCCGTTACCTAATGG; LDHA Reverse, TCCACTCCATACAGGCAC ACTGG.

### 2.6 RNA interference and gene overexpression

Small interfering RNA (siRNA) method was applied to knockdown KLHDC8A and LDHA. KLHDC8A siRNA (sequence: TGAAGGTCGTGGAGATGTA), LDHA siRNA (sequence: CTTGGAAGATAAGTGGTTT) and negative control (NC) siRNA were purchased from RiboBio. SiRNA transfection was performed with 100 nmol/L siRNA and riboFECT™ CP Transfection Kit (RiboBio) via the manufacturer's protocol. The cDNA encoding KLHDC8A was subcloned into the pTSB02‐GFP‐PURO vector. KLHDC8A overexpression plasmid was transfected into U251 cells with Lipofectamine 3000 (Invitrogen) according to the manufacturer's protocol.

### 2.7 Lactate and glucose treatment

For lactate treatment, glioma cells which incubated for 48 hours were treated with lactate (Roche) for 3 hours and harvested. For glucose treatment, glioma cells which incubated for 48 hours were starved in glucose‐free medium for 2 hours and treated with medium which containing different concentration of glucose for 3 hours. For the measurement of glucose‐induced lactate accumulation in culture medium, LA assay kit (Solarbio^®^ BC2230) was used according to instruction of the manufacturers.

### 2.8 Cell proliferation and cycle assay

The xCELLigence RTCA DP (ACEA Biosciences) was used to monitor cell proliferation in real time as previously described.^17^ Cells transfected with siRNA or plasmid were seeded in 16‐well E‐plate at 8000 cells per well. Then, the cell index was measured using xCELLigence system (ACEA Biosciences). The growth curve was analysed by Excel (Microsoft).

For the colony formation assay, cells transfected with NC or KLHDC8A siRNA for 24 hours were seeded in 6‐well plate at 2000 cells per well. About 2 weeks later, the colonies were washed with PBS and fixed in 4% paraformaldehyde for 30 minutes. Following with crystal violet staining for 15 minutes, the colonies were imaged and quantified.

For the cell cycle assay, the cells were harvested 48 hours after NC or KLHDC8A siRNA transfection and fixed in 75% ethanol at 4°C overnight. Then, the cells were incubated with 100 μg/mL RNase A and 50 μg/mL propidium iodide (PI) for 30 minutes and DNA contents were analysed in Beckman Coulter FC500MPL flow cytometer. The data were analysed by using FlowJo V10 software (FlowJo LLC).

### 2.9 Cell migration and Matrigel invasion assay

The xCELLigence RTCA DP was used for the migration assay. Cells which transfected with siRNA or plasmid were seeded in each well of the upper chamber of CIM Plate 16 at a total of 20 000 cells which resuspended in 100 μL serum‐free medium. Meanwhile, the lower chamber was added 165μl DMEM which containing 10% serum. The cell index was measured using xCELLigence system (ACEA Biosciences). The growth curve was analysed by Excel (Microsoft). Similarly, CIM Plate 16 upper chambers precoated with diluted Matrigel (356234; BD Biosciences) were used for Matrigel invasion assay. The 100 μL cells (4 × 10^5^ cells/mL) which resuspended in serum‐free medium were added to the upper chamber. Then, the lower chamber was added DMEM containing 10% serum. The cell index was measured using xCELLigence (ACEA Biosciences) and analysed by Excel (Microsoft).

Wound‐healing assay was also used for the migration assay. Cells were seeded in 6‐well plate for 24 hours with complete culture medium and transfected with NC or KLHDC8A siRNA. The cells which grown to confluence were scratched using 200 μL tip and washed with serum‐free medium. Then, a phase contrast microscope was used to capture the photographs at different time points.

### 2.10 Cell apoptosis assay

The cells were harvested after transfecting with siRNA or plasmid and washed with ice‐cold PBS twice. Then, the cells which resuspended in 300 μL of binding buffer were incubated with 5 μL annexin V‐FITC and 10 μL PI for 15 minutes in the dark at room temperature. Next, the cells were analysed by flow cytometer (Beckman Coulter FC500MPL).

### 2.11 Western blots

Expression of Bcl2 [124; Mouse mAb; Cell Signaling Technology (CST), Danvers, MA, USA], BAX (D4E4; Rabbit mAb; CST), p‐ERK (D13.14.4E; Rabbit mAb; CST), ERK (137F5; Rabbit mAb; CST), p‐P38 MAPK (D3F9; Rabbit mAb; CST), P38 MAPK (D13E1; Rabbit mAb; CST), p‐AKT (D9E; Rabbit mAb; CST), AKT (11E7; Rabbit mAb; CST), p‐STAT1 (58D6; Rabbit mAb; CST), STAT1 (D1K9Y; Rabbit mAb; CST), p‐JAK1 (D7N4Z; Rabbit mAb; CST), JAK1 (E3A6M; Rabbit mAb; CST), p‐JNK (EP1597Y; Rabbit mAb; Abcam), JNK (EPR16797; Rabbit mAb; Abcam), MMP2 (Rabbit Polyclonal; ProteinTech), P21 (Rabbit Polyclonal; ProteinTech), CDK2 (Rabbit Polyclonal; ProteinTech) was determined by Western blots as previously described with the following modifications.^18^, ^19^ Briefly, cells were lysate by Beyotime Membranous and Cytoplasmic Protein Extraction Kit via the manufacturer's protocol. Then, proteins were resolved by 10% SDS‐PAGE gel and transferred to a nitrocellulose membrane which incubated with primary and secondary antibody. Protein bands were visualized by using Tanon 5200 Chemiluminescence Imaging System. The protein band was quantified by using the ImageJ software (National Institutes of Health).

### 2.12 Statistical analyses

Data are presented as the mean ± SD of three independent experiments. All statistical were calculated in GraphPad Prism 5 by Student's *t* test to compare the data. Differences were considered statistically significant at *P < 0.05, **P < 0.01 and ***P <0 .001.

## 3 RESULTS

### 3.1 Screening for differentially expressed mRNAs in GEO database

**FIGURE 1:**
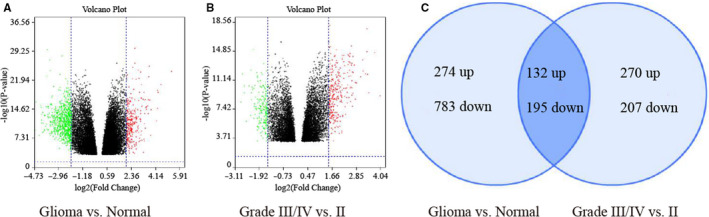
Screening of differential expression genes from the GEO database. A, Volcano plot of gene differential expression between glioma tissues with normal brain tissues. B, Volcano plot of gene differential expression between high‐grade glioma tissues (WHO grade III and IV) with low‐grade glioma tissues (WHO grade II). C, Venn diagrams represent the differentially expressed genes among glioma vs normal and grades III/IV vs II in glioma. The vertical lines correspond to|log2FC|>2 (left panel) or|log2FC|>1.5 (right panel) and the horizontal lines represent *P*‐value < 0.05. The green and red points in the plot represent the down‐regulated and up‐regulated genes with statistical significance, respectively

To examine the function of mRNAs in glioma tumorigenesis, we obtained the genome wide expression from GEO databases (GSE4290). Among the mRNAs detected by the microarray, 274 mRNAs were up‐regulated and 783 mRNAs were down‐regulated in glioma tissues compared with normal tissues with |log2FC|>2 and *P*‐value < 0.05 (Figure 1A and Table S1). Then, we analysed the expression difference of mRNAs between low‐grade glioma (WHO grade II) with high‐grade glioma (WHO grade III and IV). Using |log2FC|>1.5, *P*‐value < 0.05 as the cut‐off criteria, 270 mRNAs were up‐regulated and 207 mRNAs were down‐regulated in high‐grade glioma tissues compared with low‐grade glioma tissues (Figure 1B and Table S2). Through the intersection of these two groups of differential genes, 132 mRNAs were increased and 195 mRNAs were decreased (Figure 1C and Table S3).

### 3.2 KLHDC8A expression is increased in glioma and associated with WHO Grade

**FIGURE 2:**
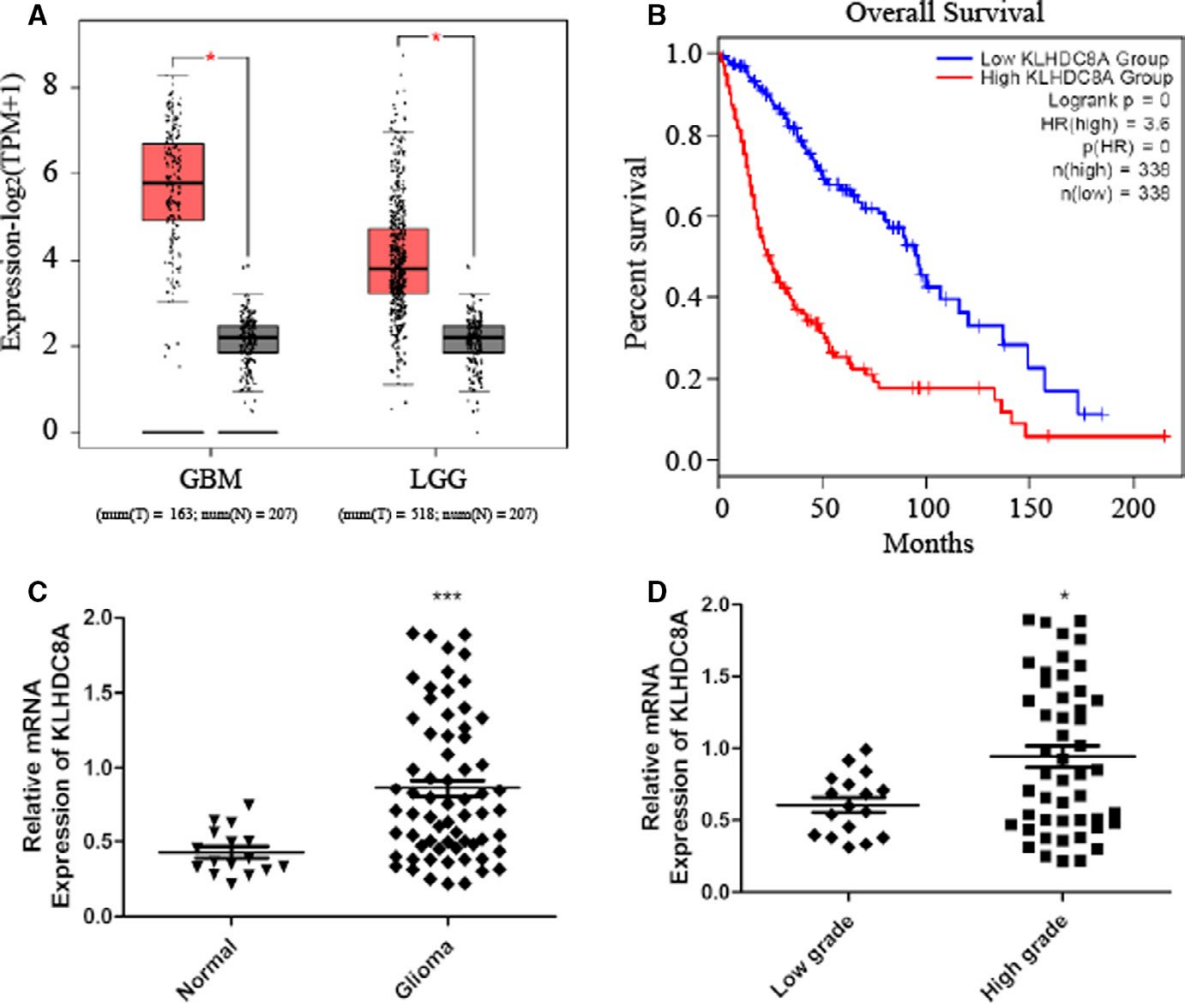
Analysis the expression of KLHDC8A in glioma. A, Expression level of KLHDC8A in GBM and LGG was higher than normal brain from GEPIA2 database. B, Survival curves were plotted for glioma (n = 676) from GEPIA2. C, KLHDC8A expression in glioma tissues was higher than normal brain tissues. KLHDC8A expression in 66 glioma tissues and 14 normal brain tissues was detected by RT‐PCR after RNA of brain tissues was extracted. D, KLHDC8A expression in high‐grade glioma (WHO grade III and IV) was higher than low‐grade glioma (WHO grade II). Data of KLHDC8A expression in glioma tissues was from C. (normal and N: non tumour samples; T: glioma; GBM: glioblastoma multiforme; LGG: low‐grade glioma), *P* values for comparisons: *P < 0.05

Using the GEPIA2 database to verify the mRNA expression and its relationship with patient overall survival, we found that the expression of KLHDC8A was significantly increased in glioma vs. controls and correlated with overall survival in patients simultaneously (Figure 2A,B). Meanwhile, the GSE2223 data set demonstrates that KLHDC8A is highly expressed in gliomas and the expression of KLHDC8A in GBM and LGG was higher than normal in GSE4290 data set, respectively (Figure S1). Then, we detected the expression of KLHDC8A in glioma and normal brain tissues collected from the Department of Neurosurgery of Yijishan Hospital of Wannan Medical College via RT‐PCR analysis. The results shown that KLHDC8A expression was up‐regulated in 66 glioma tissues as compared to the 14 normal brain tissues (Figure 2C). Further analysis indicated that KLHDC8A expression in high‐grade glioma (WHO grade III and IV) was high than low‐grade glioma (WHO grade II) (Figure 2D). These results suggest that high expression of KLHDC8A may play a role in the development of glioma.

### 3.3 KLHDC8A knockdown inhibits glioma cell proliferation, migration and invasion

**FIGURE 3:**
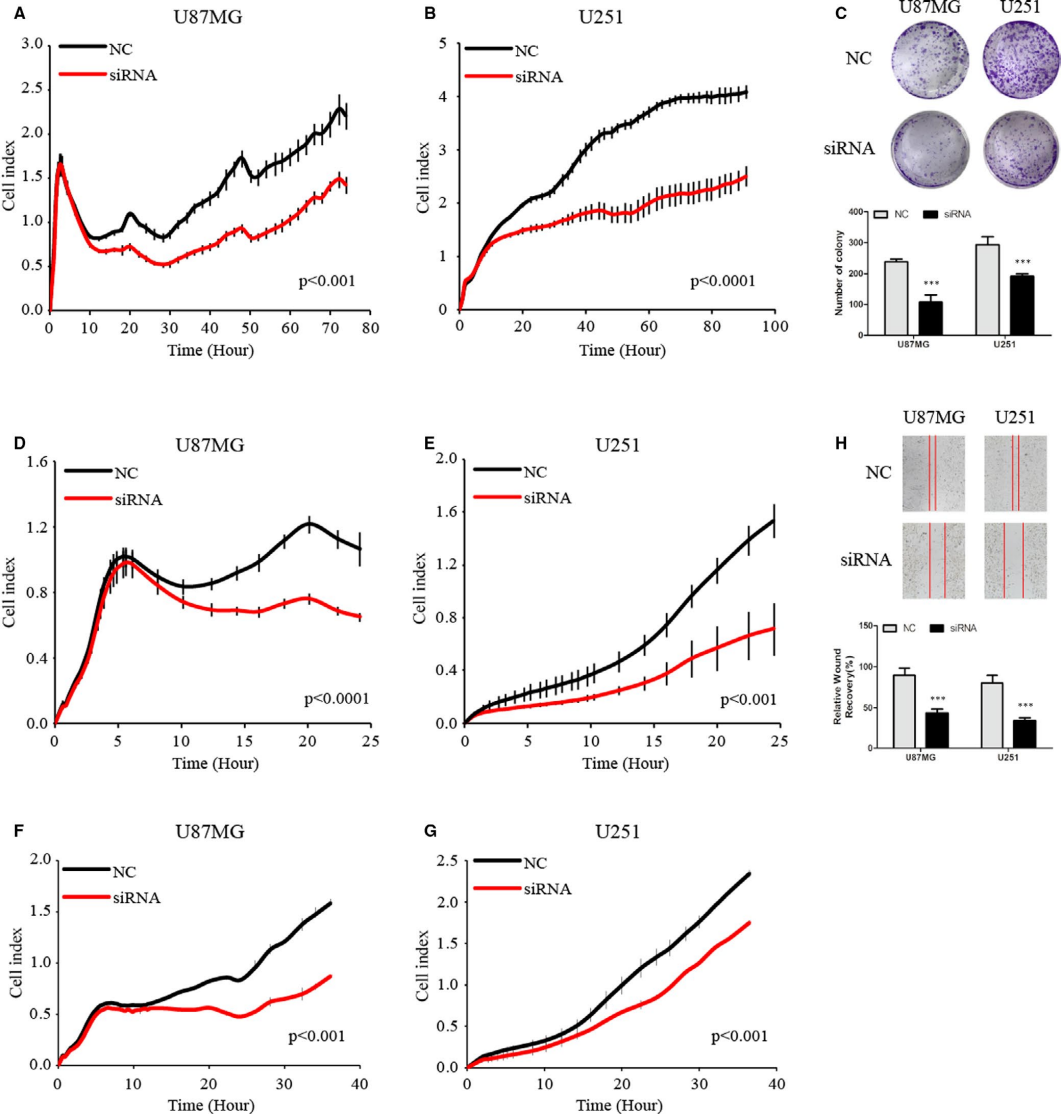
KLHDC8A regulates glioma cell proliferation, migration and invasion. A and B, KLHDC8A knockdown inhibits glioma cells proliferation. U87MG and U251 cells were transfected with NC and KLHDC8A siRNA for 24 h. Then, 8000 transfected cells per well were incubated in E‐plate and the cell index was detected by using RTCA xCELLigence at 1‐h intervals for 100 h. C, KLHDC8A knockdown inhibits the colony formation of glioma cells. U87MG and U251 cells were transfected with NC and KLHDC8A siRNA for 24 h. Then, 2000 cells per well were incubated in 6‐well plate for 2 wk. Colony was captured after paraformaldehyde fixation and crystal violet staining. D and E, Transwell assays showed that the migration of U87MG and U251 cells was significantly reduced after KLHDC8A knockdown. F and G, Transwell assays showed that the invasion of U87MG and U251 cells was significantly reduced after KLHDC8A knockdown. H, Wound‐healing assays showed that migration of glioma cells was significantly reduced after KLHDC8A knockdown. Data are mean ± SD from three independent experiments. ***P < 0.001. NC, negative control; siRNA, KLHDC8A gene silencer

To examine the function of KLHDC8A in glioma, the expression of KLHDC8A was depressed in glioma cells via siRNA transfection. The RT‐qPCR and Western blot assay shown that the mRNA level of KLHDC8A was significantly decreased in U87MG and U251 cells following KLHDC8A siRNAs transfection compared with negative control siRNA (Figure S2). Then, we used xCELLigence RTCA DP to monitor cell proliferation in real time. As shown in Figure 3A,B, glioma cell growth was inhibited when the expression of KLHDC8A was knockdown. Meanwhile, KLHDC8A knockdown also caused a decrease in glioma cell colony formation (Figure 3C). These data suggest that KLHDC8A may promote proliferation of glioma.

In addition, the effect of KLHDC8A on the migration and invasion was determined by transwell and wound‐healing assay, respectively. The vitro assessment of transwell on the glioma cells using xCELLigence RTCA DP revealed that KLHDC8A silence slow down the migration and invasion of glioma cells (Figure 3D ‐G). Meanwhile, the wound‐healing analysis also confirmed the function of KLHDC8A on cell migration (Figure 3H).

### 3.4 KLHDC8A knockdown induces cell cycle arrest and apoptosis

**FIGURE 4:**
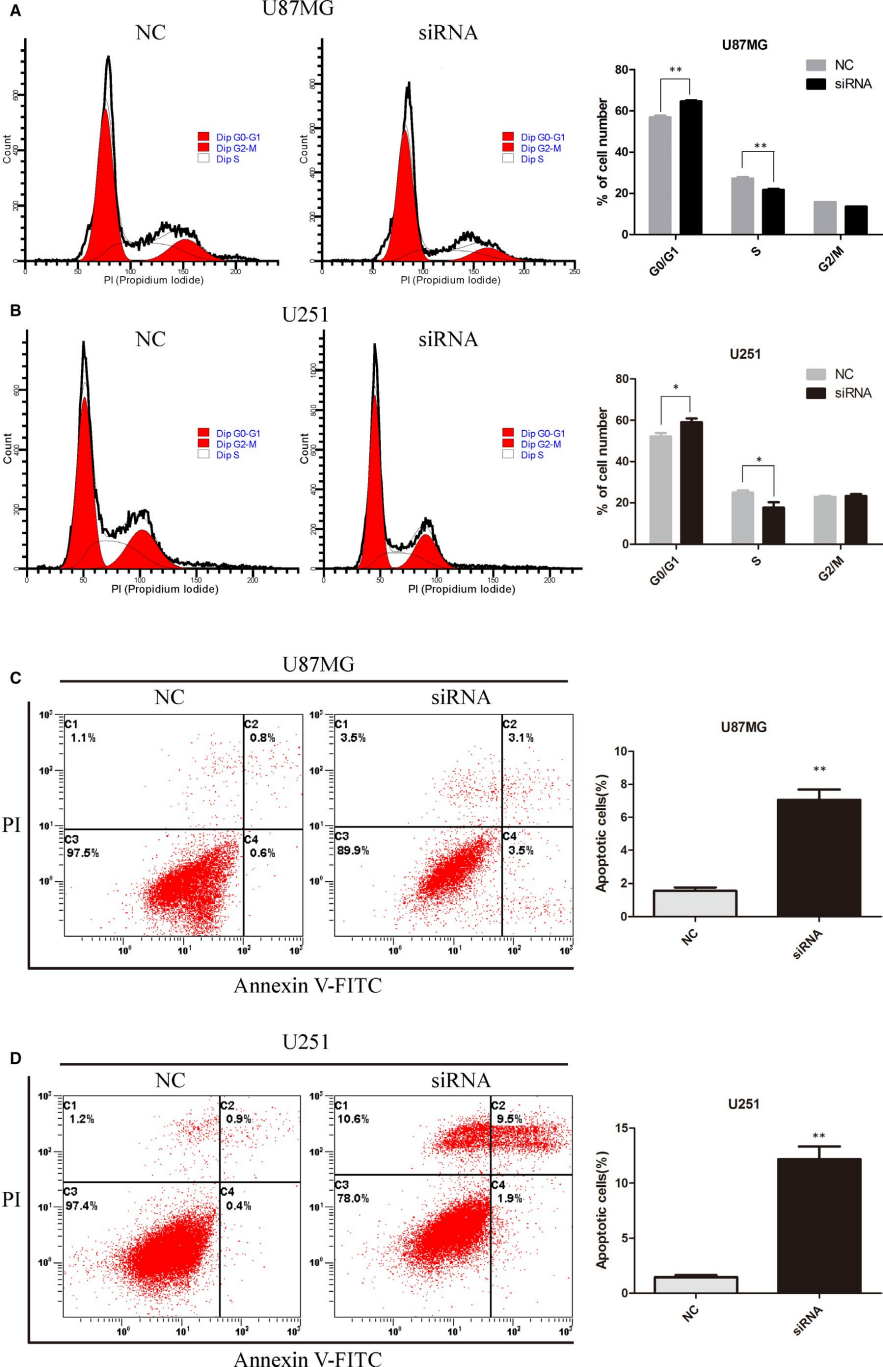
KLHDC8A regulates glioma cell cycle and apoptosis. A and B, KLHDC8A knockdown induces the G0/G1 phase arrest of glioma cells. The U87MG and U251 cells which transfected with NC and KLHDC8A siRNA for 48 h were fixed with 75% ethanol at 4°C overnight before incubated with RNase A and propidium iodide (PI) for 30 min at 37°C. Then, the DNA contents were detected by flow cytometer. The cell cycle was analysed by using FlowJo software. Histograms show the percentage (%) of cell populations at different stages of the cell cycle. C and D, KLHDC8A knockdown induces apoptosis in glioma cells. The U87MG and U251 cells which transfected with NC and KLHDC8A siRNA for 48 h were incubated with annexin V‐FITC and propidium iodide (PI) for 15 min at 4°C. Then, the cell apoptosis was detected by flow cytometer. The cell apoptosis were analysed by using FlowJo software. Histograms show the percentage (%) of cell apoptosis. Data are mean ± SD from three independent experiments. *P < 0.05, **P < 0.01. NC, negative control; siRNA, KLHDC8A gene silencer

Next, we employed flow cytometry to determine whether KLHDC8A was involved in the cell cycle. In U87MG cells, the percentage of cells in the G0/G1 phase was increased from 57.0% (NC) to 64.7% (KLHDC8A siRNA), while the S phase was decreased from 27.25% (NC) to 21.72% (KLHDC8A siRNA). Similar result had also appeared in U251 cells (Figure 4A,B). Collectively, these results indicated that KLHDC8A knockdown could affect the progression of the cell cycle by inducing the G0/G1 phase arrest.

According to previous studies, KLHDC8A promoted the proliferation. So we detected the role of KLHDC8A in apoptosis by flow cytometry after knockdown of KLHDC8A in U87MG and U251 cells. As shown in Figure 4C,D, cells transfected with NC and KLHDC8A siRNA were stained with annexin V and PI. We found that apoptotic cells of KLHDC8A siRNA group showed a significant increase compared with the NC group (Figure 4C,D). These results indicate that KLHDC8A play a role in glioma cancer cell apoptosis.

### 3.5 The target genes of KLHDC8A are identified in glioma

**FIGURE 5:**
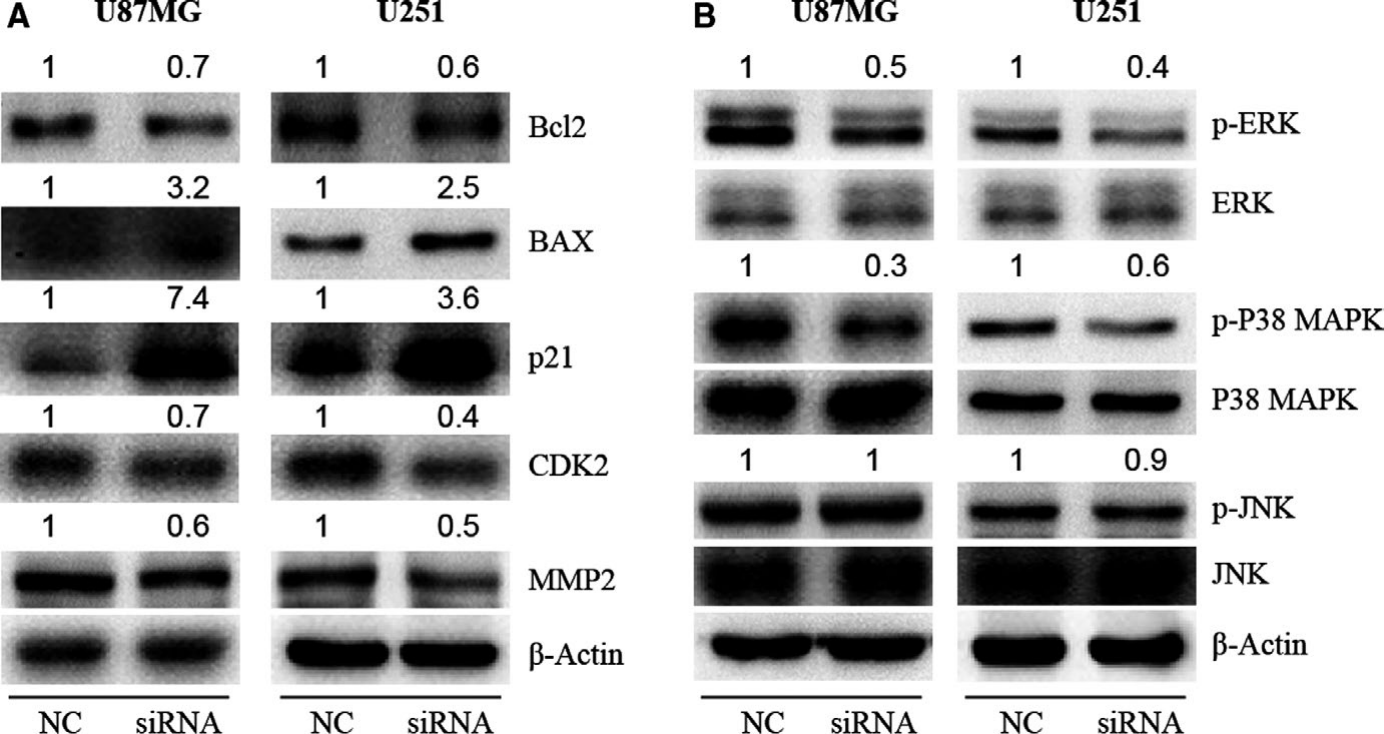
The genes regulated by KLHDC8A in glioma cells. A, Proteins related to apoptosis (BAX and Bcl2), cell cycle (p21 and CDK2) and migration (MMP2) were detected in U87MG and U251 cells after KLHDC8A knockdown. The U87MG and U251 cells which transfected with NC and KLHDC8A siRNA for 48 h were harvested. Then, Western blot detected the expression of BAX, Bcl2, p21, CDK2 and MMP2 in U251 cells. B, The levels of MAPK signalling proteins (p‐ERK, p‐P38 MAPK and p‐JNK) in KLHDC8A knockdown cells were detected. Numbers on up of bands are fold change of level relative to corresponding negative control. β‐Actin was used as loading control. NC, negative control; siRNA, KLHDC8A gene silencer

The above studies show that KLHDC8A regulated glioma proliferation, cell cycle and metastasis. To further confirm the function of KLHDC8A in cell apoptosis, the protein expression of BAX and Bcl2 which are key regulators of cell apoptosis was detected.^20^ We found that the BAX was significantly up‐regulated, whereas the Bcl2 were down‐regulated with reduced KLHDC8A (Figure 5A). Cyclin‐dependent kinase inhibitor p21 and cyclin‐dependent kinase CDK2 mediate cell cycle arrest at the G1/S.^21^ In KLHDC8A‐silenced cells, p21 was increased, whereas the CDK2 were decreased. Then, we determined the expression of MMP2 which is required for tumour metastasis.^22^ As shown in Figure 5A, MMP2 expression was significantly decreased after KLHDC8A knockdown.

We then determined the effect of KLHDC8A on multiple signalling pathways, such as MAPK, AKT and JAK‐STAT signalling pathway which play role in tumorgenesis.^23^, ^24^, ^25^ Western blot was used to detect the phosphorylated forms of MAPK family members (ERK1/2, JNK and p38 MAPK), AKT and JAK‐STAT family members (JAK1 and STAT1) in glioma cells which transfected with NC and KLHDC8A siRNA. As shown in Figure 5B, KLHDC8A knockdown inhibited the expression of the phosphorylated forms of ERK and p38 MAPK. However, the expression of activated JNK, AKT, JAK1 and STAT1 was not affected by KLHDC8A depletion (Figure 5B and Figure S3).

### 3.6 KLHDC8A overexpression regulates proliferation, migration, invasion and apoptosis, and activates ERK and p38 MAPK signalling

**FIGURE 6:**
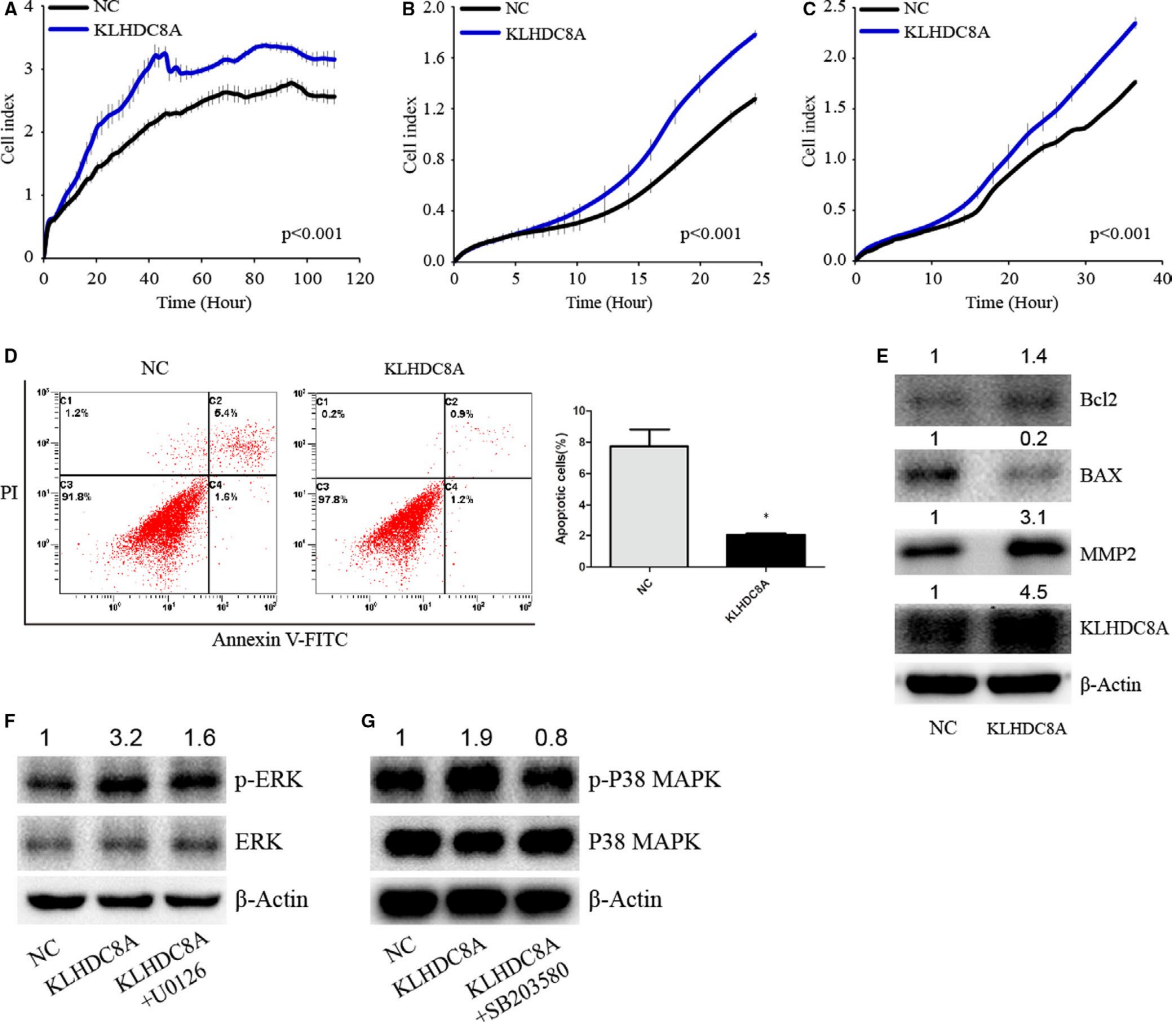
KLHDC8A overexpression regulates proliferation, migration, invasion and apoptosis, and activates ERK and p38 MAPK signalling. A, KLHDC8A overexpression promotes glioma cells proliferation. The RTCA assay was used to measure U251 proliferation ability. B and C, Transwell using RTCA revealed that KLHDC8A overexpression promotes the migration and invasion of glioma cells. D, Cell apoptosis induced by starvation in U251 were detected using flow cytometry. Histograms show the percentage (%) of cell apoptosis. E, Apoptosis‐ and migration‐related proteins were detected in U251 transfected with the NC or KLHDC8A plasmid. F, U251 were pretreated with KLHDC8A plasmid and the ERK inhibitor U0126 (10 μmol/L) for 24 h Then, Western blot examined for the protein levels. G, U251 were pretreated with KLHDC8A plasmid and the p38 MAPK inhibitor SB203580 (10 μmol/L) for 24 h Then, Western blot examined for the protein levels. Numbers on up of bands are fold change of level relative to corresponding negative control. Data are mean ± SD from three independent experiments. *P < 0.01. NC, negative control. KLHDC8A, KLHDC8A plasmid

To confirm the function of KLHDC8A in glioma, we constructed a KLHDC8A expression plasmid and transfected the plasmid into U251 cells. The results of RTCA, Matrigel invasion and apoptosis assays demonstrated that overexpression of KLHDC8A promoted glioma cell proliferation, migration, invasion and inhibited apoptosis (Figure 6A‐D). Then, we determined the associated proteins in glioma cell transfected with the KLHDC8A plasmid. The ratio of Bcl2/BAX and expression of MMP2 were increased in glioma cell with the KLHDC8A plasmid (Figure 6E). KLHDC8A overexpression also induced the phosphorylation of ERK and p38 MAPK (Figure 6F,G). When KLHDC8A overexpressed glioma cells were pretreated with ERK inhibitor U1026 and p38 MAPK inhibitor SB203580,^26^, ^27^ phosphorylation of ERK and p38 MAPK was depressed. These results suggest that KLHDC8A influences the ERK and p38 MAPK signalling.

### 3.7 Lactate and glucose induce KLHDC8A expression in glioma cells

**FIGURE 7:**
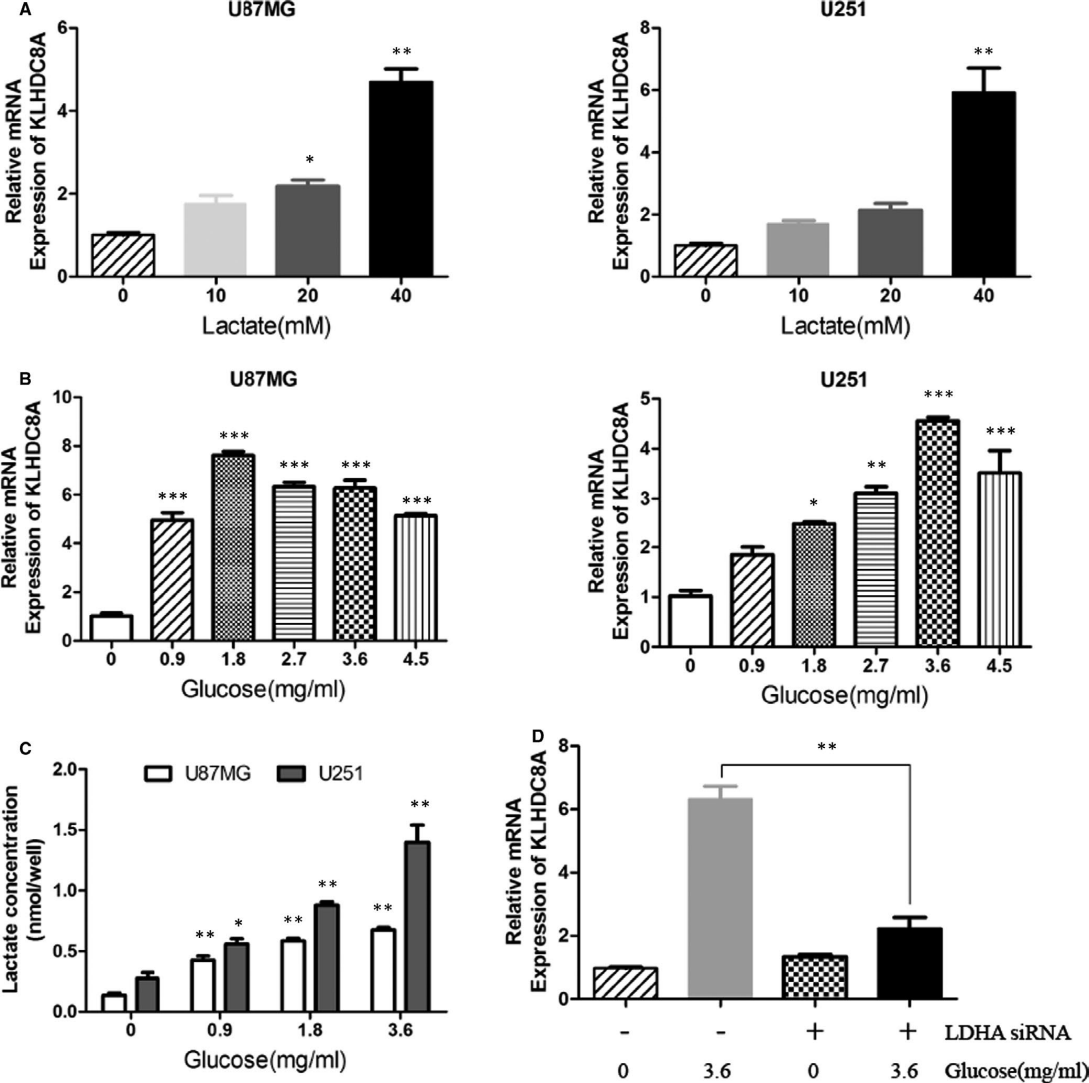
Lactate regulates KLHDC8A expression in glioma cells. A, KLHDC8A expression was increased in glioma cells following lactate (0, 10, 20 and 40 mmol/L) treatment for 3 h. RT‐PCR detected the mRNA expression of KLHDC8A in U87MG and U251. B, KLHDC8A expression was increased in glioma cells following glucose (0‐4.5 mg/mL) treatment for 3 h. RT‐PCR detected the mRNA expression of KLHDC8A in U87MG and U251. C, Lactate was accumulated following with glucose treatment. U87MG and U251 cells were stimulated with 0‐3.6 mg/mL glucose for 1 h and lactate was detected. D, LDHA knockdown decreased the expression of KLHDC8A which stimulated by glucose. U251 cells were transfected with NC and LDHA siRNA for 48 h. Then, cells were treated with 0 and 3.6 mg/mL glucose for 3 h before RT‐PCR. Data are mean ± SD from three independent experiments. *P < 0.05, **P < 0.01, ***P < 0.001

Since the function of lactate‐enriched microenvironment in tumorgenesis,^28^ we guessed that lactate might regulate the expression of KLHDC8A. RT‐PCR was performed to determine the transcripts of KLHDC8A in glioma cells which treated with different concentrations of lactate (0‐40 mmol/L). With the increase of exogenous lactate concentration, the expression of KLHDC8A was gradually increased (Figure 7A). In the tumour microenvironment, lactate accumulation was a result of increased glycolysis.^29^ We want to know whether glycolysis was correlated with KLHDC8A expression levels. As expected, the glioma cells stimulated with gradient concentrations of glucose (0‐4.5 mg/mL) showed a significant increase in the expression of KLHDC8A (Figure 7B). Meanwhile, glioma cells stimulated with glucose displayed a significant increase in the accumulation of lactate which is the judgment of glycolytic metabolism (Figure 7C). Furthermore, knockdown of LDHA, which catalyses the conversion of pyruvate to lactate in glycolysis,^30^ resulted in the decrease of glucose‐induced expression of KLHDC8A (Figure 7D and Figure S4). These results suggest that lactate which produced by glycolysis is responsible for induction of KLHDC8A expression in the tumour microenvironment.

## 4 DISCUSSION

Glioma is the most lethal and aggressive malignant tumour of the central nervous system.^31^ Although many improvements were achieved in treatment against glioma, it is still a deadly disease with frequent recurrence and poor prognosis.^32^ KLHDC8A has been proved to be highly expressed in glioma and associated tumour growth.^12^ However, the function and molecular mechanism of KLHDC8A in glioma remain need further study. In this study, overexpression of KLHDC8A in glioma tissues was confirmed by glioma samples of our hospital, as well as GEPIA and GEO databases. Knocking down and overexpressing KLHDC8A in glioma cells indicated that KLHDC8A regulated glioma tumorgenesis, such as proliferation, migration, invasion cell cycle and apoptosis. Changes in the expression of proteins related to apoptosis, cell cycle and migration in KLHDC8A‐silenced cells were also consistent with the KLHDC8A function in glioma. And the molecular mechanism of KLHDC8A in the regulation of glioma development was further investigated.

In previous studies, KLHDC8A showed highly fold increase in ΔEGFR‐deplete cells. Meanwhile, ΔEGFR knockdown significantly promoted the activation of ERK and p38 MAPK pathways.^12^ Therefore, we speculate that KLHDC8A is closely related to MAPK signal pathway. MAPK which consist of several protein kinase cascades are key signalling pathways that regulate a wide variety of cellular processes, including proliferation, stress responses, apoptosis and differentiation.^33^, ^34^ Due to genetic and epigenetic alterations, changes in MAPK signal cascades have been found in various cancer, including glioma.^35^ In this study, we determined the relationship of KLHDC8A with MAPK pathways in glioma cells. Interestingly, we found a significant decrease in the phosphorylation of both ERK and p38 MAPK after silencing of KLHDC8A. Meanwhile, KLHDC8A overexpression stimulated an obvious activation of the ERK and p38 MAPK. Our further research data showed that both U0126 and SB203580 could abolish KLHDC8A overexpression‐induced activation of ERK and p38 MAPK. Therefore, we hypothesized KLHDC8A‐promoted glioma cells proliferation, migration and invasion via activating ERK/P38 MAPK pathway.

The Warburg effect as a hallmark of cancer refers to that tumour cells preferentially perform glycolysis, even under aerobic conditions.^36^ It is characterized by high glucose uptake, active glycolysis and high lactate production in metabolites.^37^ Reprogramming of energy metabolism consequently results in high accumulation of lactate in the tumour microenvironment.^38^ Recent findings shown that lactate was positively correlated with higher incidence of metastases, tumour progression and poor prognosis.^39^ Lactate can act as a signalling molecule that activates HIF‐1α and PD‐L1 in tumour cells.^40^, ^41^ The results in our study also confirmed the relationship between lactate and tumorgenesis. We found that lactate and glucose can simulate the KLHDC8A expression in a concentration‐dependent manner. Meanwhile, the KLHDC8A expression which induced by glucose is dependent of LDHA in glioma cells.

## 5 CONCLUSION

In summary, we have shown that KLHDC8A is up‐regulated in glioma and plays role on cell proliferation, cell cycle and apoptosis as well as migration and invasion. KLHDC8A also modulates the ERK and p38 MAPK pathways. Additionally, we provide evidence for KLHDC8A induction in response to lactate and glucose stimulation. Findings in the study of KLHDC8A may provide a promising biomarker and therapy target for the treatment of glioma.

## CONFLICT OF INTEREST

The authors declare no competing interests.

## AUTHOR CONTRIBUTION

**Xiaolong zhu:** Conceptualization (lead); Data curation (lead); Formal analysis (lead); Funding acquisition (equal); Investigation (lead); Methodology (lead); Project administration (lead); Resources (lead); Software (lead); Supervision (lead); Validation (lead); Visualization (lead); Writing‐original draft (lead); Writing‐review & editing (lead). **Tianbing Chen:** Conceptualization (supporting); Data curation (supporting); Formal analysis (supporting); Funding acquisition (equal); Investigation (supporting); Methodology (supporting); Project administration (supporting); Resources (supporting); Software (supporting); Supervision (supporting); Validation (supporting); Visualization (supporting); Writing‐original draft (supporting); Writing‐review & editing (supporting). **Hui Yang:** Conceptualization (supporting); Data curation (supporting); Formal analysis (supporting); Funding acquisition (equal); Investigation (supporting); Methodology (supporting); Project administration (supporting); Resources (supporting); Software (supporting); Supervision (supporting); Validation (supporting); Visualization (supporting); Writing‐original draft (supporting); Writing‐review & editing (supporting). **Kun Lv:** Conceptualization (lead); Data curation (supporting); Formal analysis (supporting); Funding acquisition (equal); Investigation (supporting); Methodology (supporting); Project administration (lead); Resources (supporting); Software (supporting); Supervision (supporting); Validation (supporting); Visualization (supporting); Writing‐original draft (supporting); Writing‐review & editing (lead).

## Supporting information

Table S1

Table S2

Table S3

Appendix S1

## Data Availability

The data used to support the findings of this study are available from the corresponding author upon request.
