# Supplementary material for: Lactate induced up‐regulation of KLHDC8A (Kelch domain‐containing 8A) contributes to the proliferation, migration and apoptosis of human glioma cells
Source: J Cell Mol Med. 2020 Aug 26;24(20):11691–702. doi: 10.1111/jcmm.15780 (PMC7579713; doi:10.1111/jcmm.15780)

***Supplementary Information***

**Lactate induced** **upregulation of KLHDC8A (Kelch domain containing 8A) contributes to the proliferation, migration and apoptosis of human glioma cells.**

Xiaolong Zhu^1,2,3^︱Tianbing Chen^1,2,3^︱Hui Yang^1,2,3^︱Kun Lv^1,2,3^

### ^1^ Key Laboratory of Non-coding RNA Transformation Research of Anhui Higher Education Institutes (Wannan Medical College), Wuhu, 241001, PR China.

### ^2^ Non-coding RNA Research Center of Wannan Medical College, Wuhu 241001, China.

### ^3^ Central Laboratory, The first affiliated hospital of Wannan Medical College, Wuhu, 241001, PR China

**Correspondence**

Kun Lv

Email: lvkun315@126.com

**Figure legends**

**FIGURE S1.** KLHDC8A expression in the GEO database. (A) Expression level of KLHDC8A in glioma was higher than normal brain from GSE2223 database. (B and C) Expression level of KLHDC8A in GBM and LGG was higher than normal brain from GSE4290 database. (Normal: non tumor samples; Tumor: glioma; GBM: glioblastoma multiforme; LGG: low grade glioma), *P* values for comparisons: * *P* <0.05, *** *P* < 0.001.

**FIGURE S2.** RT-PCR and western blot was used to detecte the KLHDC8A expression in three siRNAs targeting KLHDC8A in glioma cells. (A) The U87MG and U251 cells which transfected with NC and KLHDC8A siRNA-1、-2、-3 for 48 h were harvested. Then RT-PCR detected the expression of KLHDC8A. (B) The U87MG and U251 cells which transfected with NC and KLHDC8A siRNA-3 for 48 h were harvested. Then western blot detected the expression of KLHDC8A. NC: negative control. siRNA: KLHDC8A gene silencer. ** *P* < 0.01, *** *P* < 0.001.

**FIGURE S3.** The levels of signaling proteins (p-AKT, p-JAK1 and p-STAT1) in KLHDC8A depleted cells were detected. β-Actin was used as loading control. NC: negative control. siRNA: KLHDC8A gene silencer.

**FIGURE S4.** RT-PCR was used to detecte the LDHA expression in three siRNAs targeting LDHA in glioma cells. The U251 cells which transfected with NC and LDHA siRNA-1、-2、-3 for 48 h were harvested. Then RT-PCR detected the expression of LDHA. NC: negative control. siRNA: LDHA gene silencer. ** *P* < 0.01.

**TABLE S1.** The differently expression genes in normal brain tissues and glioma tissues from GSE4290.

**TABLE S2.** The differently expression genes in low grade and high grade glioma from GSE4290.

**TABLE S3.** The co-differentially expressed genes among normal vs glioma and low grade vs high grade glioma from GSE4290.

**Figures**

**Supplementary Figure S1**


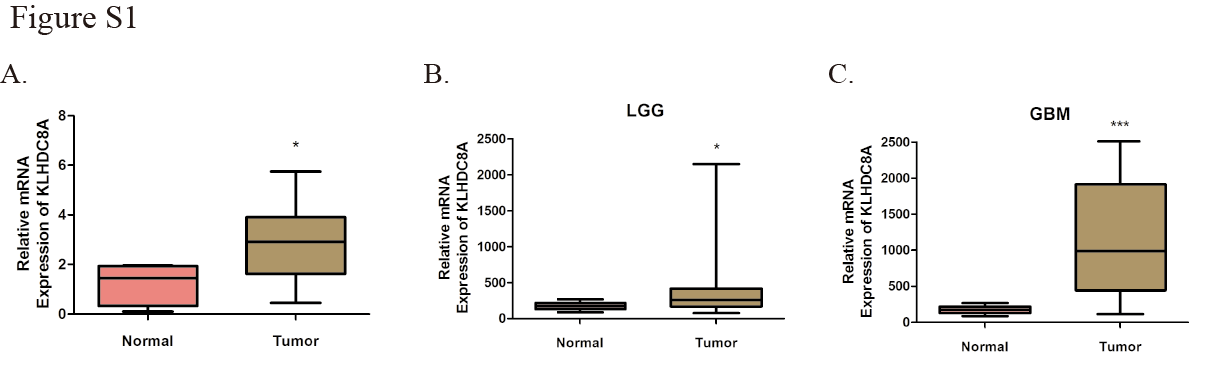


**Supplementary Figure S2**


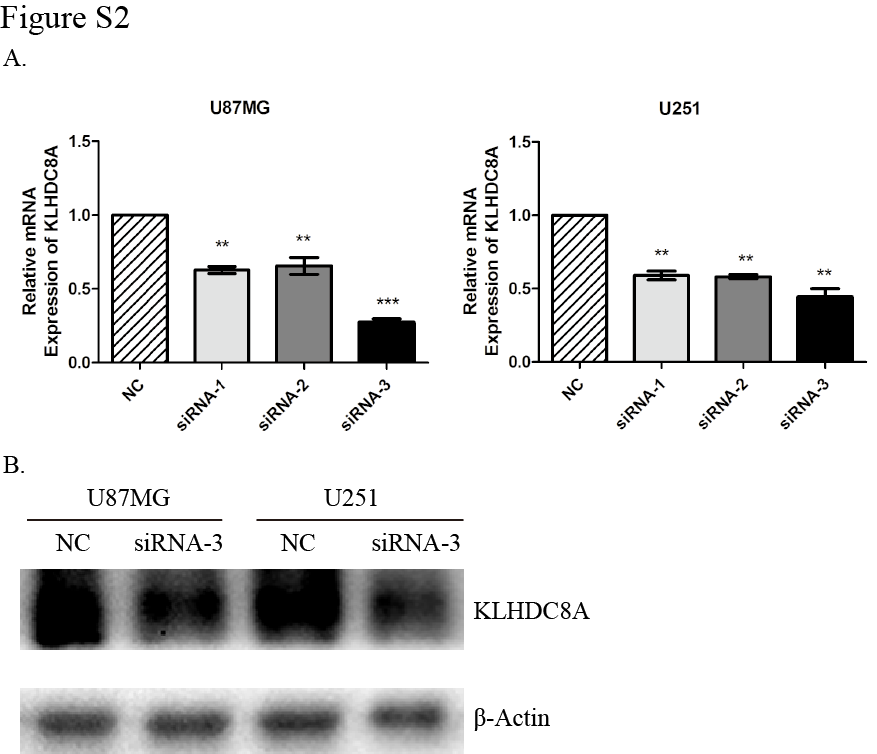


**Supplementary Figure S3**


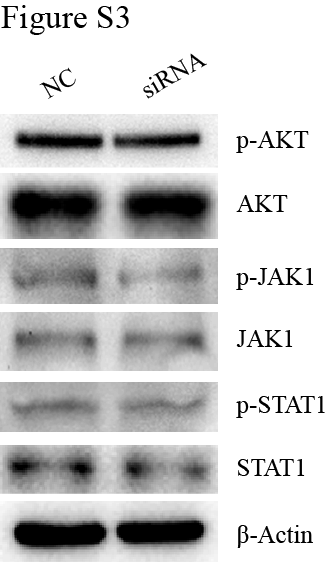


**Supplementary Figure S4**


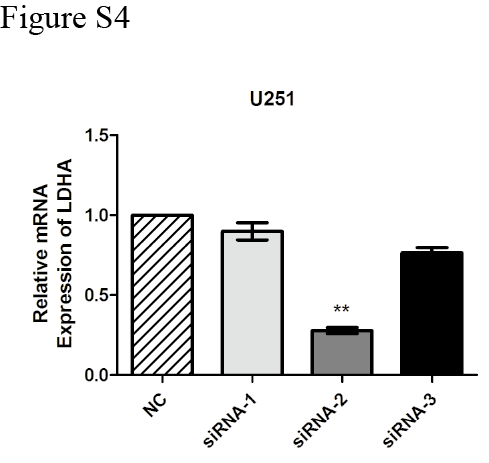

Supplement: Supplementary file 4 — Appendix S1 [file JCMM-24-11691-s004.docx]
